# Efficacy and safety of Shenqi Fuwei granule combined with perioperative regimen in the treatment of locally advanced gastric cancer: a randomized, double-blind, placebo-controlled multicenter clinical trial protocol

**DOI:** 10.3389/fonc.2026.1867721

**Published:** 2026-08-14

**Authors:** Bowen Xu, Xiaoxiao Zhang, Jiao Jiao, Qi Tang, Xingui Xiong, Zhenyang Liu, Xin He, Dongfang Li

**Affiliations:** 1 Integrative Medicine Oncology Center, The Affiliated Cancer Hospital of Xiangya School of Medicine, Central South University/Hunan Cancer Hospital, Changsha, China; 2 Department of Integrated Traditional Chinese and Western Medicine, Xiangya Hospital, Central South University, Changsha, China; 3 Digestive and Urinary Internal Medicine I, The Affiliated Cancer Hospital of Xiangya School of Medicine, Central South University/Hunan Cancer Hospital, Changsha, China

**Keywords:** event-free survival, locally advanced gastric cancer, randomized controlled trial, Shenqi Fuwei granule, study protocol, traditional Chinese medicine

## Abstract

**Background:**

While the introduction of neoadjuvant therapy has innovated perioperative treatment strategies for locally advanced gastric cancer (LAGC), preventing postoperative recurrence and metastasis remains a key clinical challenge. Previous research has indicated that Shenqi Fuwei granules (SQFWG), a Traditional Chinese medicine (TCM) prescription, possesses anti-gastric cancer properties. However, evidence from perioperative intervention studies is lacking. Our trial aims to evaluate the efficacy and safety of SQFWG combined with perioperative therapy in reducing postoperative recurrence and metastasis in LAGC, to explore clinical characteristics of the benefiting population, and to provide an evidence base for developing treatment protocols.

**Methods:**

This is a prospective, randomized, double-blind, placebo-controlled, multicenter clinical trial. We plan to recruit 286 perioperative LAGC patients from multiple hospitals in China. Participants will be randomly assigned (1:1) using a central interactive web response system with allocation concealment, stratified by the use of neoadjuvant chemotherapy ± immunotherapy, to either the control group (n=143, placebo containing 5% SQFWG) or the experimental group (n=143, SQFWG). Both groups will concurrently receive perioperative treatment (chemotherapy ± immunotherapy). Follow-up assessments will be conducted every 3 months for 3 years after treatment completion or upon reaching an endpoint event. The primary efficacy endpoint is event-free survival. Secondary outcomes include overall survival, cumulative 1-/2-/3-year recurrence and metastasis rates, surgical conversion rate, quality of life, and safety. At the same time, prespecified subgroup analyses will be performed according to the use of immunotherapy and the type of chemotherapy regimen, and the clinical applicability of SQFWG will be further explored.

**Discussion:**

To our knowledge, this is one of the first multicenter randomized double-blind placebo-controlled trials evaluating the efficacy of SQFWG in perioperative LAGC. This trial is expected to provide high-level evidence for the role of TCM in perioperative comprehensive management of LAGC.

**Clinical trial registration:**

## 1 Introduction

Gastric cancer (GC) ranks as the fifth most common malignant tumor globally, estimated 968,400 new cases and 659,900 deaths, making it the second most incidence and the third most mortality in the digestive system (1). The incidence and mortality of GC in China surpass the global average, accounting for 37.04% of new cases and 39.46% of deaths worldwide (2). Due to its non-specific early symptoms, approximately 70% of Chinese patients are diagnosed at an advanced stage. While gastrectomy with D2 lymphadenectomy is the standard surgical treatment for locally advanced gastric cancer (LAGC) (3), about 40-60% of LAGC patients experience postoperative recurrence and metastasis, resulting in a 5-year survival rate below 50% (4). Landmark phase III trials, such as CLASSIC (5), ARTIST (6), RESOLVE (7), RESONANCE (8), PRODIGY (9), and MATTERHORN (10), have established the role of perioperative therapy (neoadjuvant and adjuvant chemotherapy). These regimens improve tumor shrinkage, clinical downstaging, R0 resection rates, and survival outcomes while reducing recurrence, thereby solidifying the perioperative treatment model. Despite these advances, 40.6-48.9% of patients still experience recurrence within three years post-surgery, and 39-47.9% die within five years (4).

The advent of immune checkpoint inhibitors has revolutionized the treatment landscape for advanced GC, spurring investigation into their synergistic potential with chemotherapy in the perioperative setting, as seen in trials like KEYNOTE-585 (11), DRAGON IV (12), and notably MATTERHORN (10). The MATTERHORN study demonstrated the potential of chemoimmunotherapy in resectable LAGC, showing an 8% improvement in 2-year EFS (67% vs. 59%) and a 6.7% improvement in 3-year OS (68.6% vs. 61.9%). Mechanistically, chemotherapy can induce immunogenic cell death and deplete immunosuppressive cell populations in the tumor microenvironment, thereby potentially enhancing the efficacy of concomitant immunotherapy (13, 14). Furthermore, preoperative immunotherapy may prime systemic anti-tumor immunity, while its continuation postoperatively could help eradicate minimal residual disease, collectively reducing recurrence risk. This underpins the “chemo-reduction plus immune-consolidation” strategy evaluated in MATTERHORN, where durvalumab combined with FLOT chemotherapy aimed to shrink tumor burden preoperatively and eliminate residual cells postoperatively (15). Preventing recurrence and metastasis remains a critical bottleneck for improving the prognosis of LAGC, and the introduction of immunotherapy has underscored the urgent need for novel therapeutic strategies.

Accumulating evidence indicates that Traditional Chinese Medicine (TCM) offers unique advantages in cancer prevention, attenuating treatment-related toxicity, enhancing therapeutic efficacy, and reducing recurrence and metastasis (16–19). Only one meta-analysis focusing on postoperative adjuvant chemotherapy combined with TCM for LAGC reported extended DFS by approximately 2.61 months (mean difference [MD]: 2.61 months, 95% CI [1.38, 3.83]), reduced the cumulative 1-, 2-, 3-, and 5-year recurrence/metastasis rates by 39%, 40%, 51%, and 40%, alongside reduced chemotherapy-related toxicities such as myelosuppression and gastrointestinal reactions (20). Furthermore, a recent multicenter prospective cohort study showed that the TCM formula Yiqi Huayu Jiedu Decoction reduced the postoperative recurrence/metastasis rate by 34.7% and improved the 5-year survival rate by 14.24% (85.29% vs. 71.05%) in stage III LAGC (21). This underscores the significant therapeutic potential of TCM as a complementary modality to reduce postoperative recurrence and metastasis in LAGC. However, evidence specifically in the perioperative LAGC setting remains limited, and there is a notable lack of clinical trials evaluating the continuous administration of TCM throughout the entire perioperative period. High-quality evidence is still needed to determine whether integrating TCM into contemporary perioperative therapy for LAGC can effectively reduce recurrence and prolong survival.

The Chinese herbal compound WeiFuFang, developed by Professor Pan Minqiu, a National Master of Chinese Medicine in the Department of Integrated Traditional Chinese and Western Medicine at Hunan Cancer Hospital, was optimized into Shenqi Fuwei Granules (SQFWG) after dosage form improvement. This formulation was registered as a hospital-prepared TCM product with the Hunan Medical Products Administration (Registration No. Xiangyao Preparation Z20240984000). Modern pharmacological studies suggest its active components possess anti-angiogenic, immunomodulatory, and pro-apoptotic properties (22–31). Preliminary clinical studies indicate that this compound could prolong median survival, improve survival rates, enhance quality of life (QoL), and exhibits a favorable safety profile in patients with LAGC (32–35). However, robust evidence from large-scale, multicenter clinical trials is lacking regarding whether SQFWG could improve perioperative outcomes, reduce postoperative recurrence and metastasis, alleviate symptoms, and enhance QoL in LAGC within the contemporary perioperative therapy framework. Therefore, our trial represents the first multicenter effort to evaluate the integration of TCM compound with perioperative therapy, aims to determine the clinical efficacy and safety of SQFWG combined with neoadjuvant/adjuvant chemotherapy ± immunotherapy in reducing postoperative recurrence and metastasis in LAGC.

## 2 Methods

### 2.1 Study design

**Figure 1:**
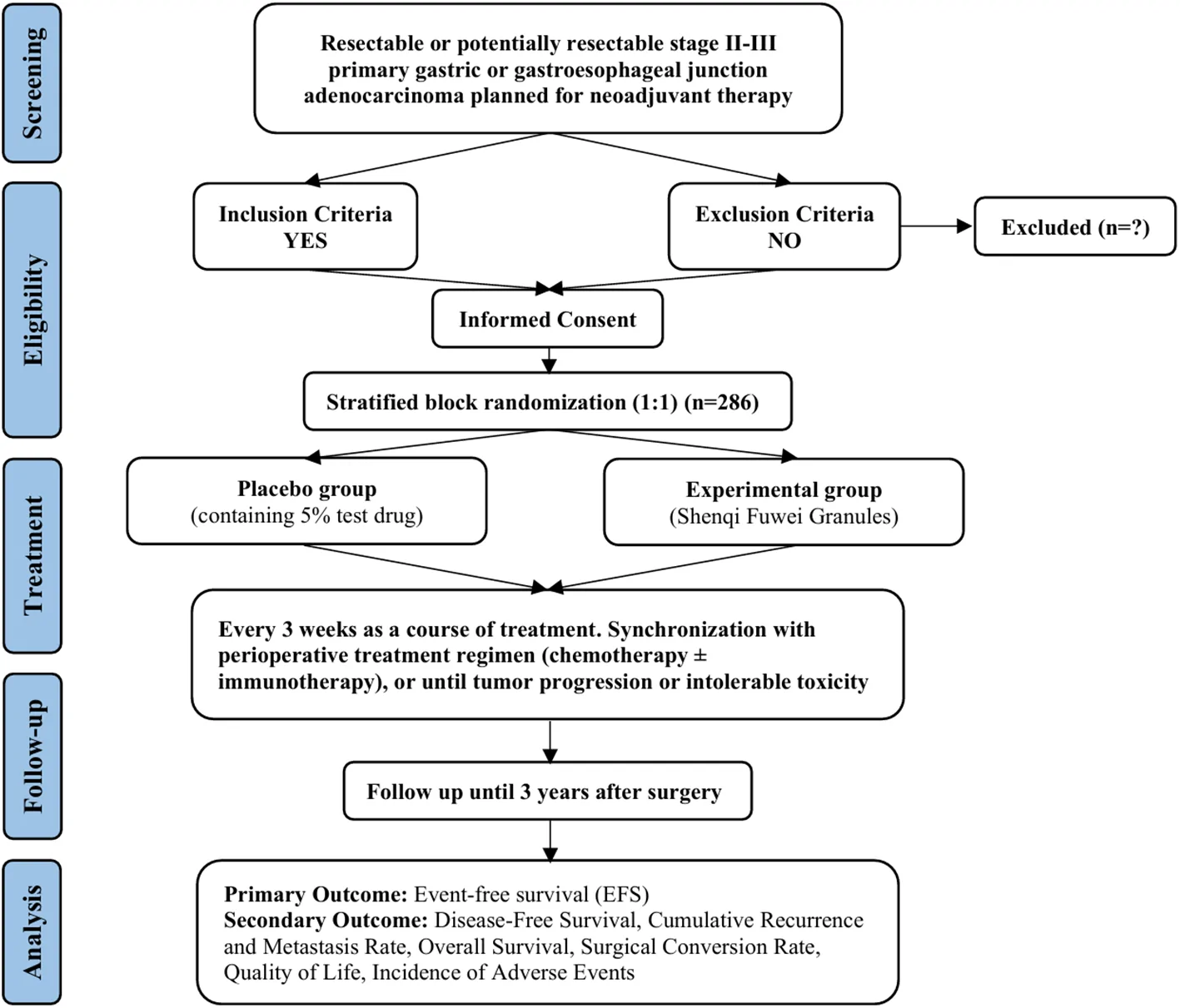
Flowchart of treatment phases.

**Figure 2:**
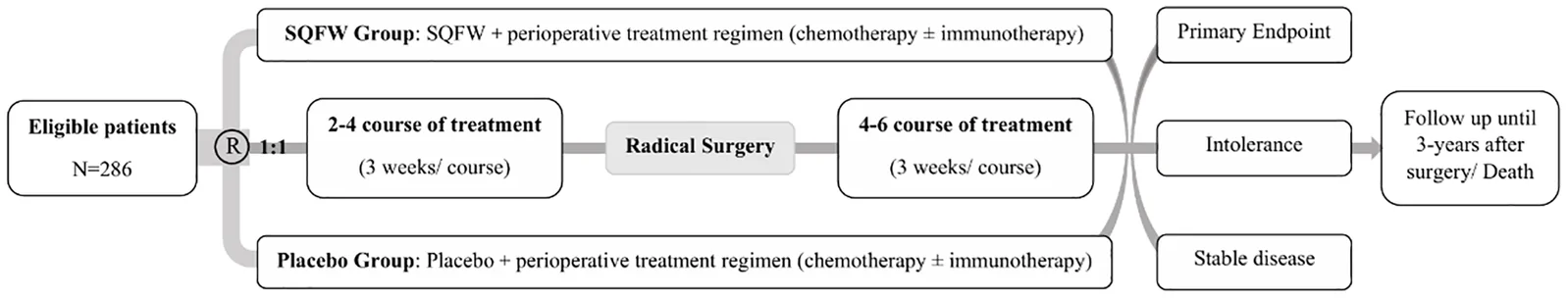
Intervention and follow-up flow chart.

This study is a multicenter, randomized, double-blind, placebo-controlled clinical trial conducted in China. It will be implemented across at least seven tertiary hospitals, including Hunan Cancer Hospital, Xiangya Hospital of Central South University, Guangdong Provincial Hospital of Traditional Chinese Medicine, Jiangsu Provincial Hospital of Traditional Chinese Medicine, the First Affiliated Hospital of Hunan University of Traditional Chinese Medicine, and Guangxi International Zhuang Medical Hospital. Central randomization with allocation concealment will be employed, stratified by the use of neoadjuvant chemotherapy ± immunotherapy. All participants will receive perioperative chemotherapy (SOX or XELOX) ± immunotherapy. In addition, patients in the experimental group will receive SQFWG, while those in the control group will receive a matched placebo containing 5% SQFWG. The study medication will be administered concurrently with chemotherapy and continued until disease progression, unacceptable toxicity, or completion of the perioperative treatment phase. All patients will be followed for 3 years. After completing the final treatment or upon reaching a study endpoint, patients will enter a follow-up phase with assessments scheduled every 3 months. The primary efficacy endpoint is event-free survival (EFS). **Figure 1** presents the flowchart for participant enrollment, intervention, and assessment. **Figure 2** illustrates the schedule of interventions and follow-up. The study phases are outlined in **Table 1**.

**Table 1:** The schedule of participants.

Project			Lead-in period	Intervention period						Follow-up period ^[2]^
				Preoperative visit (2 ~ 4 cycles)		Operation	Postoperative visit (4 ~ 6 cycles)			
Timepoint			V0	V1	VN		Postoperative V1	Postoperative V2	Postoperative VN	N+V
Visit date			-W1-0W	W3 ± 1W	WN*3 ± 1W	Date of operation	Postoperative ± 4W	WN*3 ± 1W	WN*3 ± 1W	Every 3 months ± 1M
Inclusion/exclusion criteria			✓							
Signed informed consent			✓							
Collected medical history										
demographic data			✓							
diagnosis			✓							
Past history and concomitant diseases			✓							
Therapeutic observation										
Vital signs			✓	✓	✓		✓	✓	✓	*
Physical examination			✓	✓	✓		✓	✓	✓	*
Physical condition score			✓	✓	✓		✓	✓	✓	✓
MDASI-TCM			✓	✓	✓		✓	✓	✓	
Description of tongue and pulse			✓	✓	✓		✓	✓	✓	
TCM syndrome			✓	✓	✓		✓	✓	✓	
Imaging Examination^[1]^	CT		✓	Repeated every 2–3 courses during treatment						*
	MRI, B-ultrasound		*							*
	Bone scanning		*							*
	Gastroscope		*							*
Tumor marker			✓	✓	✓		✓	✓	✓	*
FACT-Ga			✓	✓	✓		✓	✓	✓	
ESAS			✓	✓	✓		✓	✓	✓	
Infections and Complications Assessment ^[3]^							✓			✓
Safety observation										
Routine blood/ urine/stool			✓	✓	✓		✓	✓	✓	*
Liver/kidney function			✓	✓	✓		✓	✓	✓	*
ECG			✓	✓	✓		✓	✓	✓	*
NCI-CTCAE				✓	✓	✓	✓	✓	✓	✓
AEs				✓	✓	✓	✓	✓	✓	✓
Biological Sample Collection										
Biological Sample Collection		blood	✓			√ Preoperative Collection			√ (End of treatment/Progression)	
		urine								
		stool								
		Tongue coating								
		Pathological tissue				✓				
Other work										
Drug combination				✓	✓		✓	✓	✓	✓
Efficacy evaluation				✓	✓		✓	✓	✓	✓
Medication compliance			✓	✓	✓		✓	✓	✓	✓

* in the table indicates that this check is optional. ^[1]^For imaging examinations, the same examination method should be used for screening of the same site and for each review after enrollment, and the examination should be reviewed every 2–4 sessions during the treatment phase and every 3 months during the follow-up phase. Head, chest, and abdominal examinations should be selected as appropriate. If CT/MRI examination has been done for the same site, abdominal X-ray examination and ultrasound examination are not necessary. Bone scanning examination is selected according to the patient's condition. ^[2]^Follow-up period: The entry nodes were ① completion of established perioperative treatment (chemotherapy ± immunotherapy), ② disease progression, and ③ intolerable toxicity, and the above conditions were selected. The follow-up period was based on the subject 's hospital selection for in-hospital visits/telephone visits. Vital signs, physical examinations, and other ancillary tests are optional and may not be done at telephone visits. If the subject completes relevant auxiliary examinations in another hospital, the examination results should be collected. [3] Assessment of infection and complications: Complications were assessed only in the early (30 days) and late (12 months) postoperative period.

MDASI-TCM, M.D. Anderson Symptom Traditional Chinese Medicine. TCM, Traditional Chinese Medicine. CT, Computed Tomography. MRI, Magnetic Resonance Imaging. FACT-Ga, Functional Assessment of Cancer Therapy - Gastric. ESAS, Edmonton Symptom Assessment System. ECG, Electrocardiogram. NCI-CTCAE, National Cancer Institute-Common Terminology Criteria for Adverse Events. AEs, Adverse Events.

### 2.2 Participant screening and selection

The diagnosis of LAGC will be established based on the Chinese Society of Clinical Oncology guidelines for GC (2024). Pathological classification will follow the World Health Organization Classification of Digestive System Tumors, 5th edition, and tumor staging will adhere to the AJCC Cancer Staging Manual, 8th edition. Potentially eligible patients meeting these diagnostic criteria will be systematically assessed against the specific inclusion and exclusion criteria detailed below. Patients who fail to meet all inclusion criteria or meet any exclusion criterion will not be enrolled in the study.

#### 2.2.1 Inclusion criteria

1. Histologically or cytologically confirmed adenocarcinoma of the stomach or gastroesophageal junction.
2. Resectable or potentially resectable disease with a clinical stage of cT1-2bN1-3M0, cT3-4bN0M0, or cT3-4aN1-3M0 (Stage II–III).
3. Previous anti-tumor treatment, including chemotherapy, radiotherapy, immunotherapy or targeted therapy, is not permitted;
4. Adequate organ function, defined as:
  • Absolute neutrophil count ≥ 1.5 × 10^9^/L
  • Hemoglobin ≥ 9.0 g/dL
  • Platelet count ≥ 80 × 10^9^/L
  • Serum albumin ≥ 30 g/L
  • Serum total bilirubin ≤ 1.5 × upper limit of normal (ULN)
  • Aspartate aminotransferase and alanine aminotransferase ≤ 2.5 × ULN
  • Alkaline phosphatase ≤ 2.5 × ULN
  • Serum creatinine ≤ 1.5 × ULN
5. Karnofsky Performance Status score ≥ 70.
6. Age between 18 and 75 years.
7. Willing and able to provide written informed consent.

*Potentially resectable disease is defined, as clinical stage II–III disease, according to CSCO gastric cancer guidelines and multidisciplinary team (including surgical oncologists and radiologists) assessment, including tumors considered technically unresectable at diagnosis but potentially convertible to R0 resection following neoadjuvant therapy.

#### 2.2.2 Exclusion criteria

1. Evidence of distant metastasis.
2. Known hypersensitivity or contraindication to fluorouracil, oxaliplatin, or any component of the investigational Chinese herbal product.
3. Combined with other parts of the primary malignant tumor. Subjects without disease for 5 years, or with a history of completely resected non-melanoma skin cancer or successfully treated carcinoma *in situ* were eligible for the study;
4. Acute gastrointestinal complications such as complete/incomplete obstruction, active bleeding, perforation, or dysphagia that would preclude oral intake.
5. Pregnancy or lactation. Patients with severe neurological, psychiatric, or cognitive disorders that would impair compliance or consent.
6. Significant cardiac disease within 6 months prior to enrollment, including uncontrolled angina, arrhythmia, congestive heart failure, myocardial infarction, or cardiac insufficiency.
7. Any other concurrent severe acute or chronic medical or psychiatric condition, or laboratory abnormality that, in the investigator’s judgment, would increase the risk associated with study participation or interfere with the interpretation of study results.
8. Legal incompetence or any other condition rendering the patient unsuitable for study participation.

#### 2.2.3 Criteria for discontinuation of study intervention

1. Occurrence of an adverse event or intercurrent illness preventing the completion of at least 4 treatment cycles.
2. Voluntary withdrawal of informed consent by the subject.
3. Pregnancy during the study or loss to follow-up.

#### 2.2.4 Criteria for study withdrawal

1. Subject’s request to withdraw from all study-related procedures.
2. Poor protocol compliance as judged by the investigator.
3. Termination of the study by the sponsor.
4. Regulatory mandate to halt the trial.
5. Any other reason deemed necessary by the investigator for the subject’s well-being or the integrity of the study.

#### 2.2.5 Management of discontinuations/withdrawals

The investigator will make every reasonable effort to retain participants in the study and on the assigned treatment, unless discontinuation is considered to be in the subject’s best interest. For any subject who discontinues the study intervention or withdraws from the study, the investigator will attempt to perform and document an end-of-treatment assessment.

For participants who discontinue study intervention due to an adverse event or toxicity, safety follow-up will continue for at least 28 days after the last dose of study medication (or until resolution or stabilization of the AE, whichever is longer). All AEs occurring during this period will be recorded and graded. For participants who withdraw consent or are lost to follow-up without an ongoing AE, only survival follow-up will be performed as described above. Survival follow-up will be conducted every 3 months (telephone contact is permissible) until the occurrence of a study endpoint event.

### 2.3 Randomization method

The blinded document (Participant Randomization Table and Drug Assignment Table) of this trial is generated by unblinded statistician using SAS software (Version 9.4) and uploaded to the central randomization system by the randomization system administrator (provided by Beijing Bio-Thera Information Technology Co., Ltd). SAS software was used to generate random numbers and treatment groups corresponding to random numbers. Parameters such as procedure, seed number, block length during randomization were kept in an unblinded folder and the randomization table was reproducible. These persons were not involved in participant recruitment, treatment, or outcome assessment. The central randomization system will complete the assignment of participant randomization number and drug management during the trial. This trial was stratified by neoadjuvant chemotherapy ± immunotherapy using stratified block randomization. Authorized study personnel at each participating site performed subject assignment via the randomization system after confirming eligibility and obtaining informed consent. The grouping process was done automatically by a centralized system and did not rely on the subjective judgment of the researcher.

To ensure data confidentiality and prevent unauthorized access, the randomization system maintains a complete electronic audit trail documenting all allocation-related activities, including login attempts, randomization requests, data entries, and emergency unblinding, with timestamps and user identification. Access to the randomization module is strictly role-based, with different authorization levels assigned to investigators, study coordinators, statisticians, and system administrators. The integrity of the allocation process is further overseen by independent clinical monitors. The randomization schedule itself remains sealed and inaccessible to all study personnel until database lock, except for emergency unblinding.

### 2.4 Blinding and emergency unblinding

The investigational product (SQFWG) and the placebo are identical in appearance, packaging, taste, and smell. All study medications will be manufactured, packaged, and supplied by Tiandi Hengyi Pharmaceutical Company. A unique randomization code will serve as the participant identifier. Subjects, investigators, caregivers, outcome assessors (including imaging evaluators), and data analysts will all be blinded to group assignment. Emergency unblinding of a participant’s treatment assignment via the central randomization system is permitted only if the investigator deems that knowledge of the treatment is essential for the clinical management of a serious adverse event. The system will automatically log all emergency unblinding requests and actions. The investigator must consult with the study’s principal coordinator and formally notify the trial sponsor within 24 hours following unblinding.

### 2.5 Sample size calculation

This is a randomized, placebo-controlled superiority trial with a 1:1 allocation ratio. The primary endpoint is EFS. Based on the KEYNOTE-585 trial results (11), the assumed 3-year EFS rate for the control group (receiving perioperative chemotherapy ± immunotherapy) is 44%. We hypothesize that the addition of SQFWG will increase the 3-year EFS rate by 20% (absolute improvement), resulting in a rate of 64% for the experimental group. Using a two-sided significance level (α) of 0.05 and a power (1-β) of 80%, and assuming a constant accrual rate over an enrollment period of 36 months, the sample size was calculated using PASS software (version 15). This calculation yielded 114 participants per group. To account for an estimated 20% dropout rate, the final target enrollment is set at 143 participants per group. The design is event-driven, requiring a sample size of 286 cases. The duration of enrollment is 3 years, and the total study duration is 5 years, taking into account a 20% dropout rate.

### 2.6 Interventions

- Experimental Group: Participants will receive SQFWG, composed of *Astragalus membranaceus* (Huangqi), *Codonopsis pilosula* (Dangshen), *Atractylodes macrocephala* (Baizhu), *Gallus domesticus* (Jineijin, endothelium corneum of chicken gizzard), stir-fried *Hordeum vulgare* (Chao Maiya), stir-fried *Setaria italica* (Chao Guya), *Glycyrrhiza uralensis* (Gancao), and other herbs in specified ratios, in addition to the perioperative therapy. The granules will be administered orally at a dose of one packet three times daily, dissolved in hot water and taken after meals. Each treatment cycle is 3 weeks, synchronized with the chemotherapy schedule. Administration will continue concurrently with perioperative therapy until disease progression, unacceptable toxicity, or completion of the prescribed therapy. For patients undergoing surgery, oral administration will resume postoperatively based on recovery status.
- Control Group: Participants will receive a matched placebo in addition to the perioperative therapy. The placebo is designed to be indistinguishable from the active granules in outer packaging, dosage form (color, shape), and taste. Its composition includes: SQFWG extract powder (5%, sub-therapeutic dose), maltodextrin, a bitter flavoring agent, sucralose, yellow dextrin, and brown dextrin. Maltodextrin and sucralose conform to the standards for pharmaceutical excipients as per the Chinese Pharmacopoeia (2025 Edition, Volume IV). The bitter flavoring agent, yellow dextrin, and brown dextrin are food-grade additives compliant with national food safety standards. The dosing regimen (dose, frequency, and route) is identical to that of the experimental group.
- Perioperative Therapy (Both Groups): Participating centers will follow unified protocol-specified perioperative treatment principles based on contemporary CSCO and international guidelines. Based on the results of the KEYNOTE-585 (11) and RESOLVE (7) trials, the primary chemotherapy regimens are limited to SOX or XELOX. The preoperative phase will consist of 2–4 cycles of neoadjuvant SOX or XELOX chemotherapy, with or without immunotherapy. Postoperatively, participants will receive 4–6 cycles of adjuvant SOX chemotherapy (which may be followed by S-1 monotherapy) or 4–6 cycles of XELOX chemotherapy, with or without immunotherapy. Treatment will continue until disease progression, unacceptable toxicity, or completion of the planned cycles. The specific immunotherapy regimen is not restricted by the protocol. Immunotherapy (e.g., pembrolizumab, durvalumab, camrelizumab, or other PD-1/PD-L1 inhibitors) is permitted only for patients with biomarker evidence of potential benefit (e.g., PD-L1 CPS ≥5, MSI-H, or EBV positivity) based on current guidelines. The choice of agent is at the investigator’s discretion, the specific agent, number of cycles, and timing must be documented, but not restricted to reflect real-world practice.
  ① Chemotherapy regimens:
  SOX regimen (3-week cycle): Oxaliplatin 130 mg/m², administered intravenously over 2 hours on Day 1; S-1 40–60 mg (based on body surface area: <1.25 m²: 40 mg; 1.25–1.5 m²: 50 mg; >1.5 m²: 60 mg), taken orally twice daily after meals on Days 1–14, followed by a 7-day treatment-free interval.
  XELOX regimen (3-week cycle): Oxaliplatin 130 mg/m², administered intravenously over 2 hours on Day 1; Capecitabine 1,000 mg/m², taken orally twice daily on Days 1–14, followed by a 7-day treatment-free interval.
  ② Immunotherapy regimens:
  Pembrolizumab: 200 mg administered intravenously over 30 minutes on Day 1 of each 3-week cycle.
  Durvalumab: 1,500 mg administered intravenously over 60 minutes on Day 1 of each 4-week cycle.
  Camrelizumab or Sintilimab: Administered per standard institutional protocols at approved doses (typically 200 mg Q3W for Camrelizumab; 200 mg Q3W for Sintilimab).
  The protocol specifies the recommended dosing schedules for commonly used PD-1/PD-L1 inhibitors administered according to the approved product labeling and current clinical guidelines. The duration of immunotherapy is also defined in accordance with routine perioperative treatment recommendations.
  ③ Dose adjustment rules:
  Dose modifications for chemotherapy are based on the severity of adverse events per NCI-CTCAE v5.0, including hematologic toxicity, thrombocytopenia, peripheral neuropathy, hepatic dysfunction, renal impairment, and other significant adverse events. For oxaliplatin-related neurotoxicity (grade ≥2), oxaliplatin may be held or reduced by 25%; for grade ≥3, oxaliplatin is discontinued. For thrombocytopenia (platelet <75×10^9^/L), oxaliplatin is held until recovery to ≥100×10^9^/L; for grade 3–4 thrombocytopenia, the dose is reduced by 25% upon re-administration. For S-1 or capecitabine, dose adjustments are based on gastrointestinal toxicity, hand-foot syndrome, and myelosuppression, using standard dose reduction levels (100% → 75% → 50% → discontinuation).
  Immunotherapy dose modifications follow current international consensus guidelines and the prescribing information for each immune checkpoint inhibitor, with corticosteroid management for immune-related adverse events as per standard practice, e.g., hold for grade 2 pneumonitis/colitis; discontinue permanently for grade 3 or higher. All dose modifications will be recorded prospectively.”
- Concomitant Medications: Supportive care will be provided to all patients according to clinical oncology guidelines. The use of other Chinese herbal decoctions with purported similar effects, anti-tumor Chinese herbal injections, Chinese patent medicines, thymic peptides, or other immunomodulatory biological agents is prohibited during the study period. Any changes in concomitant medications will be recorded.

### 2.7 Outcome measures

#### 2.7.1 Primary outcome

- EFS: Defined as the time from randomization to the first occurrence of any of the following events: disease progression precluding curative surgery, local or distant recurrence, or death from any cause.

#### 2.7.2 Secondary outcomes

1. Survival Outcomes:
  • DFS: Time from the date of surgery to tumor recurrence, metastasis, or death from any cause.
  • Overall Survival (OS): Time from randomization to death from any cause.
  • Cumulative 1-, 2-, and 3-Year Recurrence/Metastasis Rate: The proportion of patients experiencing recurrence or metastasis at 1, 2, and 3 years from randomization.
  • Cumulative 1-, 2-, and 3-Year Survival Rate: The proportion of patients alive at 1, 2, and 3 years from the date of surgery.
2. Surgical Conversion Rate: Proportion of patients who successfully undergo R0 resection following neoadjuvant therapy.
3. Infection and Complications:
  • Perioperative infection rate.
  • Incidence of early (≤30 days) and late (≤12 months) postoperative complications.
4. Patient-Reported Outcomes:
  • QoL: Assessed using the Functional Assessment of Cancer Therapy - Gastric scale.
  • Symptom Burden: Assessed using the Edmonton Symptom Assessment System.
  • TCM Symptoms: Assessed using the MD Anderson Symptom Inventory - TCM Module.
5. Treatment Delivery Indicators:
  • Chemotherapy Completion Rate: Proportion of planned adjuvant chemotherapy cycles completed.
  • Medication Adherence: Calculated based on the number of study drug packets consumed. Adherence will be categorized as <80%, 80-120%, and >120%. The number and percentage of patients in each category will be reported.
6. Immunological Biomarkers: Levels of circulating T cells, regulatory T cells, CD4+ T cells, and CD8+ T cells.
7. Serum Tumor Markers: Carcinoembryonic Antigen, Carbohydrate Antigen 72-4, Carbohydrate Antigen 19-9, and Carbohydrate Antigen 125.

#### 2.7.3 Safety evaluation

Adverse events will be monitored throughout the intervention period and graded according to the National Cancer Institute Common Terminology Criteria for Adverse Events, version 5.0. Laboratory assessments (including routine blood tests, liver and kidney function) and electrocardiograms will be performed at scheduled visits. The incidence of adverse drug reactions will be calculated as the number of patients experiencing any adverse drug reactions divided by the total number of enrolled patients.

### 2.8 Data collection and management

Source data will be collected using electronic case report forms within a secure, web-based Electronic Data Capture (EDC) system. Site investigators will be responsible for data entry. All study personnel will receive standardized training on the study protocol and data entry procedures.

Data management will be facilitated by a multi-center research collaboration platform - a validated EDC system hosted by Beijing Bioknow Information Technology Co., Ltd. This platform provides secure data collection, tracking, validation, and export functionalities.

Two independent clinical research coordinators will perform double data entry. The EDC system will then compare entries for verification. Following this, investigators will review and confirm the data. All data changes will be logged in the system’s audit trail. Participants will be identified by unique codes to ensure confidentiality. The principal investigator will bear overall responsibility for data integrity.

An independent Data Monitoring Committee (DMC) will periodically review data quality and safety, which empowered to recommend modifications to the trial (dose adjustment, sample size re-estimation) based on interim analyses of accrued data. The study database will be locked after resolving all data queries and completing final participant follow-up.

### 2.9 Statistical analysis

All analyses will be performed using SAS software (version 9.4). Efficacy analyses will be conducted on both the Full Analysis Set (intention-to-treat principle) and the Per-Protocol Set. Safety analyses will be performed on the Safety Set. Prespecified subgroup analyses based on randomization stratification factors, and multivariable adjustment will be used to minimize the influence of treatment heterogeneity. Time-to-event endpoints (EFS, DFS, OS) will be summarized using Kaplan-Meier survival curves and compared between groups using the log-rank test. The Cox proportional hazards model will be employed for multivariate analysis to adjust for potential confounding variables. All statistical tests will be two-sided, with a *p*-value < 0.05 considered statistically significant.

Subgroup analyses: To explore potential beneficiary characteristics, we will perform prespecified subgroup analyses according to baseline clinical factors (age, sex, tumor clinical stage, T stage, N stage, PD-L1 CPS, MSI/MMR status, chemotherapy regimen, immunotherapy use, baseline Karnofsky score, TCM syndrome classification, and relevant biomarker subgroups). For each subgroup, the treatment effect on EFS will be estimated, and interactions between treatment and subgroup factors will be tested using the Cox model. The model selection strategy will be forced entry of these pre-specified variables (no stepwise selection) to avoid overfitting and multiplicity issues. Proportional hazards assumption will be tested using Schoenfeld residuals. These analyses are exploratory and hypothesis-generating; no adjustment for multiple testing will be applied. This study is not powered for formal predictive model development or validation.

Multiple correction strategy: To control the type I error rate due to multiple secondary endpoints, we will apply a hierarchical testing procedure. The primary endpoint EFS will be tested first at α=0.05 (two-sided). Only if the primary endpoint is statistically significant will secondary efficacy endpoints (OS, DFS, 1-/2-/3-year recurrence/metastasis rates) be tested sequentially using a fixed-sequence procedure to preserve the overall α level. If the primary endpoint is not statistically significant, all secondary endpoints will be considered exploratory and reported descriptively without formal hypothesis testing. All other secondary endpoints (postoperative complications, symptom burden, TCM symptoms, immunological biomarkers, and serum tumor markers) will be reported descriptively with nominal *p*-values. These analyses are hypothesis-generating and will not be subject to formal multiplicity adjustment. Results will be interpreted with appropriate caution.

## 3 Trial status and progress

Funding sources: the Noncommunicable Chronic Diseases–National Science and Technology Major Project (Grant Nos. 2024ZD0521301 and 2024ZD0521300), 2024.12-2028.11.

Recruitment start date: November 1, 2025; expected completion: August 30, 2028.

Expected primary analysis completion: December 2030.

Expected primary results publication: anticipated in 2031 after completion of follow-up.

Number of recruited participants at submission: 61 (as of 2026.7.9).

Data collection is ongoing at the time of protocol submission; no data analysis has been performed.

## 4 Discussion

This trial protocol aims to answer the following primary question: LAGC patients receiving perioperative chemotherapy (± immunotherapy), does the addition of SQFWG improve EFS compared with placebo? We hypothesize that SQFWG will reduce postoperative recurrence and metastasis by modulating immune function and tumor metabolism. The expected findings include a clinically meaningful improvement in 3-year EFS, acceptable safety, and identification of patient subgroups that may derive greater benefit. This protocol emphasizing that results are not yet available, and the statements herein reflect the planned hypothesis and expected outcomes, to test a hypothesis, not to confirm efficacy.

This high recurrence in LAGC is closely associated with postoperative minimal residual disease, an immunosuppressive tumor microenvironment, and immune escape mechanisms, highlighting the limitations of perioperative chemotherapy alone for achieving curative control. Consequently, exploring novel combination therapies with synergistic effects is a crucial strategy for mitigating recurrence and metastasis in LAGC. In this context, the role of TCM in modulating immunity and exerting anti-tumor effects in GC is increasingly recognized.

This view is strongly supported by a recent meta-analysis demonstrating that adding *Atractylodes macrocephala*-containing TCM to neoadjuvant chemotherapy for LAGC significantly improves tumor response. Specifically, this combination increased the objective response rate by 41% (RR: 1.41) and the disease control rate by 20% (RR: 1.20), while also enhancing quality of life (MD: 8.47) and reducing chemotherapy-induced adverse reactions (36). Notably, the analysis revealed that such TCM regimens increased the proportions of peripheral CD3+, CD4+, and CD8+ T-cells, suggesting a reversal of the immunosuppressive tumor microenvironment, a key factor in preventing postoperative recurrence and metastasis in LAGC. These robust findings underscore the therapeutic potential of TCM as an adjunct to modern perioperative chemotherapy, particularly for enhancing immune function and treatment efficacy. Aligned with this evidence, our study introduces SQFWG, a refined hospital preparation derived from the original Chinese herbal compound Weifufang compound developed by Professor Pan Minqiu, and all preclinical and clinical studies cited under the name Weifufang refer to the same fundamental formula.

Our prior research has systematically evaluated the clinical value and potential mechanisms of this formula. Clinical evidence indicates that Weifufang combined with chemotherapy significantly prolongs OS by 3.23 months (13.33 vs. 10.1) in LAGC, improves 1- to 3-year survival rates, enhances QoL (assessed by the QLQ-C30 questionnaire), alleviates cancer-related fatigue (Piper Fatigue Scale), and reduces myelosuppression and gastrointestinal reactions (32–35). Preclinical studies *in vitro* and *in vivo* have revealed that its antitumor effects may involve the regulation of multiple signaling pathways. The formula can upregulate the expression of PPARγ and PTEN while downregulating proliferation-related proteins such as PTTG, c-Myc, and hTERT, thereby inhibiting GC cell proliferation and inducing apoptosis. Additionally, it suppresses the HIF-1α/VEGF pathway, suggesting anti-angiogenic properties (22–29). Network pharmacology analyses (30) predicted that multiple active components of Weifufang may act on targets including TP53, STAT3, and Akt. Subsequent *in vitro* experiments confirmed its ability to inhibit the PI3K/Akt pathway, leading to suppressed cancer cell proliferation. Non-targeted metabolomics (31) further demonstrated that Weifufang inhibits tumor growth in SGC-7901 subcutaneous xenograft models, an effect associated with the modulation of key metabolic pathways such as arginine/proline, nicotinate/nicotinamide, and phenylalanine/tyrosine/tryptophan metabolism, indicating that amino acid metabolic reprogramming is a potential mechanism of action.

Therefore, integrating SQFWG into the perioperative chemotherapy regimen represents a logical next step to test whether its immunomodulatory and metabolic effects can translate into improved EFS, reduced postoperative recurrence, and better outcomes for LAGC patients. We will conduct that multicenter, large-sample, prospective randomized controlled trial to obtain more clinical evidence-based data. By analyzing patient serum samples and building on previously identified differential metabolites, we aim to elucidate the molecular mechanisms through which this formula modulates tumor metabolism and to clarify its pharmacodynamic material basis.

Therefore, our clinical trial is designed to evaluate the clinical efficacy of integrating TCM into perioperative therapy for reducing postoperative recurrence and metastasis in LAGC. Key strengths of the trial design include: (1) the use of an Interactive Web Response System for centralized randomization with allocation concealment, which balances unmeasured confounders across multiple participating centers; (2) implementation of blinding for participants, investigators, outcome assessors, and statisticians to minimize placebo effects, information bias, and performance bias, thereby enhancing data validity; (3) administration of the TCM intervention throughout the entire perioperative period alongside chemotherapy ± immunotherapy, with strict control of concomitant medications; and (4) The use of placebo (5% SQFWG) at low concentration of the original drug is in accordance with the “*Criteria for Simulated Effect Evaluation of Placebo in Chinese Patent Medicine*” (T/CACM 1420-2022) issued by the Chinese Society of TCM, which can ensure the feasibility of ethics and trial implementation. We have nevertheless acknowledged that residual biological activity cannot be completely excluded and that such activity would be expected to bias the results toward the null hypothesis, resulting in a more conservative estimate of treatment efficacy. If any minimal biological activity exists, a positive finding would be even more robust. Furthermore, we will conduct a *post-hoc* blinding assessment using the Bang Blinding Index; if substantial unblinding occurs, we will perform sensitivity analyses to evaluate potential bias. In terms of sample size, this is a large expected effect for an adjunctive herbal intervention in the context of modern perioperative therapy, we will interpret clinical effect results with greater caution. We acknowledge that our prior studies of Weifufang (the precursor formulation of SQFWG) were small, single-center, and predominantly non-randomized, and thus provide hypothesis-generating rather than confirmatory evidence.

This protocol outlines a scientifically rigorous, clinical trial. By implementing a multicenter enrollment strategy in China and employing EFS as a robust primary endpoint, the study aims to evaluate the clinical efficacy and safety of SQFWG combined with neoadjuvant chemotherapy ± immunotherapy in reducing postoperative recurrence and metastasis in LAGC. It further seeks to identify patient subgroups most likely to benefit and to explore the underlying mechanisms of efficacy. The findings are expected to provide evidence-based support for integrating TCM into the comprehensive perioperative management of LAGC, refine its role within modern treatment strategies, optimize the application framework for herbal formulations, and establish a foundation for precise clinical use.

## Data Availability

The original contributions presented in the study are included in the article/supplementary material, further inquiries can be directed to the corresponding author.
